# How Does the Pre-Registration Midwifery Programme Prepare the Newly Qualified Midwives for Their Post-Registration Perinatal Mental Health Role? A Mixed Methods Study

**DOI:** 10.3390/healthcare12232329

**Published:** 2024-11-21

**Authors:** Yemi Onilude, Omorogieva Ojo, David Evans, John Crowley, Priti Chopra, Gordon Ade-Ojo, Kate Knightly-Jones

**Affiliations:** 1 School of Health Sciences, University of Greenwich, London SE9 2UG, UK; o.onilude@greenwich.ac.uk (Y.O.); d.t.evans@greenwich.ac.uk (D.E.); j.j.crowley@greenwich.ac.uk (J.C.); 2 School of Education, University of Greenwich, London SE9 2UG, UK; p.chopra@greenwich.ac.uk (P.C.); g.o.ade-ojo@greenwich.ac.uk (G.A.-O.); 3 Specialist Perinatal Mental Health Midwife, Darent Valley Hospital, Kent DA2 8DA, UK; kate.knightly-jones@nhs.net

**Keywords:** newly qualified midwives, midwifery education, perinatal mental health, student midwives

## Abstract

Objective: In the United Kingdom (UK) and most countries worldwide, midwives are professionally required to undertake an initial perinatal mental health (PMH) risk assessment at every maternity contact. However, studies have found that midwives feel that they are not well-equipped to provide effective care for women with PMH needs. This study explores how the newly qualified midwives (NQMs) are prepared through pre-registration midwifery education and placements to have sufficient confidence in their *knowledge*, *attitude*, *skills*, *and habits* (KASH) for their post-registration PMH role. Methods: This explanatory sequential mixed methods study collected survey data from two independent groups: NQMs (*n* = 50), who qualified from 10 UK universities, and senior specialist midwives (SSMs) (*n* = 32). Descriptive and inferential responses were analysed using SPSS. Statistical differences between the ranged Likert scale responses of the NQMs and SSMs were analysed using the Mann-Whitney U test. The *p*-value of <0.05 was considered statistically significant. The semi-structured interview phase comprised of NQMs (*n* = 12) and SSMs (*n* = 8). The qualitative data were thematically analysed using NVivo. Results: The pre-registration midwifery programme significantly prepared the NQMs to have sufficient confidence in their *knowledge* of the related PMH role, multidisciplinary team (MDT) role, and available services (*p* < 0.05) and good *attitude* towards women with varying PMH conditions (*p* < 0.0005). The NQMs had sufficient confidence in their *skills* in using the validated tool for PMH assessment, to build rapport to facilitate disclosure, and recognise deteriorating PMH (*p* < 0.01). They had regular *habits* of discussing PMH well-being at booking and made prompt referrals (*p* < 0.05). The NQMs were not prepared to have sufficient knowledge of PMH medications, perinatal suicide prevention, and the impact of maternal mental health on partners (*p* < 0.01) including children (*p* < 0.05); skills in managing PMH emergencies (*p* < 0.05), and to regularly discuss suicidal thoughts (*p* < 0.01), issues of self-harm, and debrief women following pregnancy or neonatal losses and traumatic births (*p* < 0.05). Some aspects were either confirmed or contradicted at the interviews. Conclusions and recommendations: The pre-registration midwifery programme prepares the NQMs to some extent for their post-registration PMH role. Perceived areas for improvement suggest implications for the development of educational, practice, policy, and preceptorship to facilitate the NQMs’ sustainable confidence in their KASH.

## 1. Introduction

Worldwide, perinatal mental health (PMH) conditions are mostly manageable when promptly identified, and they are neither signs of parental weakness nor inability to cope, hence, no mother or father is immune, regardless of their social-demographic or economic status [1,2,3]. Although evidence suggests that some childbearing women or birthing people have a higher risk of developing PMH conditions than others [4,5,6,7], there have been reports of the emergence of new onset without previously identified risk factors or worsening presentations in pre-existing conditions, requiring prompt management [8,9]. In addition, the maternity population in the United Kingdom (UK) is also increasingly becoming complex due to changing health profiles including widening socio-economic determinants associated with PMH needs [10,11,12]. These reports have emphasised the importance of adequate training for all maternity care practitioners including midwives on how to effectively risk assess women for PMH issues at every maternity contact to prevent avoidable complications, thus improving maternal, fetal, and infant outcomes [13,14].

Midwives are internationally identified as indispensable links working in partnership with childbearing women across the entire continuum of care from preconception, antenatal, birth, and postnatal periods from hospitals to the community, even in challenging settings or conditions [14,15]. They are distinctively positioned within the multidisciplinary team to make essential contributions to health promotion, illness prevention, and swift recovery promotion throughout the continuum of childbirth alongside considering the partner and the entire family’s needs [16,17]. At the point of midwifery qualification and joining the Nursing and Midwifery Council (NMC) register, midwives must demonstrate meeting the standards of proficiency representing the knowledge, skills, and attributes to effectively risk assess, anticipate, and recognise changes that could lead to complications and be able to provide the best and safe care for women and their families [17]. At the start of their midwifery career, the newly qualified midwives (NQMs) are expected to be prepared through pre-registration midwifery education and placements to practice safely and proficiently for all childbearing women and infants with complications including those with mental health needs [17,18].

Maternity antenatal booking appointments are usually undertaken by midwives in the UK [19] recommends that midwives ask women about their mental well-being at their booking visit and risk assess maternal mental well-being at every consultation during pregnancy and postpartum. This booking appointment includes obtaining a full current and past mental health history, and not just depression [20]. Following the initial PMH risk assessment, the booking midwife is expected to co-ordinate the individualised care planning and management of women identified at high risk or have pre-existing mental health issues by making prompt referrals to multidisciplinary and specialist teams [19].

The assessment of the midwives and student midwives’ PMH education is a well-researched area using varying research methodologies to assess gaps in knowledge, skills, competencies, and attitude on the management of women with PMH issues [21,22,23,24]. However, despite previous research on PMH education and practice focussing on midwives and future midwives [21,22,23,24], there are limited studies solely focussing on the application of the fourfold educational framework to explore the newly qualified midwives’ (NQMs) PMH preparedness, which [25] recommended for school-teachers to explore the intellectual *knowledge*, *attitudes* to this knowledge, the *skills* for dealing with it, and the routine *habits* of addressing the issues (KASH). Therefore, to address this proposed area of the NQMs’ preparedness, this current study uniquely adapted the fourfold KASH educational framework to explain the extent to which the pre-registration midwifery programme prepares the NQMs to have sufficient confidence in their *intellectual knowledge* of PMH and related issues, *attitudes* to PMH as part of their role and supporting women, the *skills* for dealing with different relevant tasks, and the routine *habits* for addressing PMH.

This study’s research question is “How well does the PMH education and placements on the pre-registration midwifery programme develop the NQMs’ *knowledge*, *attitude*, *skills and habits* in preparation for their post-registration role in working with women and birthing people at risk of or experiencing PMH issues?” This study aimed to explore the extent to which pre-registration midwifery PMH education and placements prepare the NQMs’ KASH for their post-registration role in supporting women with PMH needs. The objectives were to explore what is perceived as useful and relevant for the NQMs’ preparedness and what is perceived as not preparing the NQMs well, thus requiring improvement.

## 2. Materials and Methods

### 2.1. Study Design

**Figure 1:**
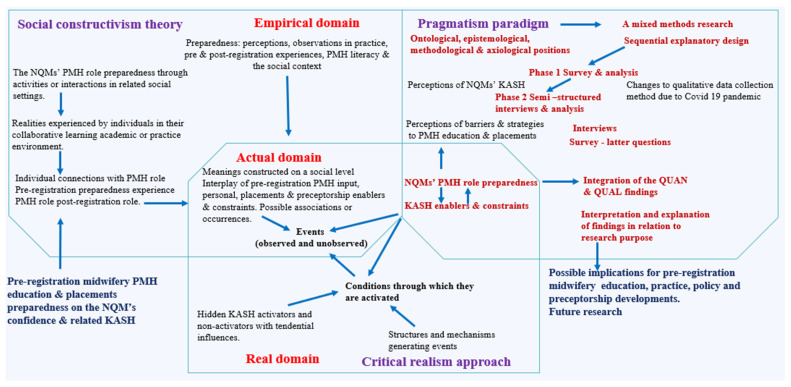
The interconnections of social constructivism, critical realism, and pragmatism to explain the NQMs’ KASH and impact on PMH role preparedness [26].

This was a sequential explanatory mixed methods design performed between September 2019 and July 2020. The applicable theoretical framework presents a dualism of social constructivism and critical realism linked to pragmatism to explain the NQMs’ KASH and the pre-registration midwifery preparedness for an effective post-registration PMH role (see the interconnections illustrated in Figure 1 [26]). Similarly, the related ontological, epistemological, methodological, and axiological perspectives were considered to address the multifaceted phenomena around the NQMs’ KASH and impacts on the PMH role. The process of integrating the two phases started with quantitative data collection and the analysis phase, which subsequently informed the qualitative data collection and the qualitative analysis phase. This latter approach was used to explain the quantitative results in more depth.

### 2.2. Setting and Sampling

The participants were recruited from two National Health Service (NHS) participating Trusts in England: South-East London and North Kent regions. These two NHS Trusts were selected because of the local population of people they serve, the ethnic diversity, location in the inner city, associated wider and complex inter-related determinants of health, their psychosocial health needs, and the additional impacts on maternal mental health, well-being, and obstetric outcomes.

### 2.3. Participants and Recruitment

The survey participants were selected using purposive sampling based on the judgement of their typical characteristics as related to the aim of the study. Survey participants formed two independent groups: newly qualified midwives (NQMs) and senior specialist midwives (SSMs).

#### Inclusion and Exclusion Criteria

Inclusion criteria:

- *NQMs* (*Group* 1): Midwives who had completed the pre-registration BSc Midwifery programme within a year before recruitment to this study, had undertaken PMH education as part of the midwifery training, registered with the Nursing and Midwifery Council (NMC) in the UK, and currently practising with the National Health Service (NHS).
- *SSMs* (*Group* 2): Midwives who were specialist PMH midwives, preceptorship lead midwives, midwifery practice facilitators, senior midwifery colleagues in specialist roles, and working in the NHS. This group also included senior midwives who particularly supported the NQMs to consolidate their practice and were able to provide information on the NQMs’ ability to support women with PMH needs.

Exclusion criteria:

- For Group 1: Midwives who were no longer newly-qualified because they had completed their BSc Midwifery programme over a year ago, those who did not undertake PMH education as part of their midwifery training, NQMs not working with the NHS; and newly qualified adult or mental health nurses providing either surgical or perinatal mental healthcare within the maternity services.
- For Group 2: Midwives who were not senior specialist midwives and not in the position to provide information on the NQMs’ ability to support women with PMH needs and non-midwifery healthcare professionals in senior or specialist posts.

The total number of NQMs and SSMs who met the selection criteria from the two participating NHS Trusts was 98.

The information for completing the survey was distributed using the research gate-keepers within each of the participating NHS Trusts to the midwives’ NHS emails, face-to-face contact in staff meetings, and preceptorship training. In light of the sensitivity of mental health issues, which some potential participants may find distressing, links to supportive mental health services available on the NHS and other mental health organisations were provided.

The ethical approval was obtained on 30 June 2019 from the Greenwich University Research Ethics Committee (UREC): *reference UREC/18.5.5.18.* This initial ethics’ application was approved for the data collection methods, using a survey for phase 1 and a face-to-face focus group for phase 2. Due to the impacts of the global COVID-19 pandemic and the subsequent lock down, it was not possible to have either group 1 or group 2 participants together for the focus group discussion. The participants also preferred video-based online semi-structured interviews via Microsoft Teams platform. A modified ethics’ application was therefore made to UREC with changes from focus group to online semi-structured interviews, along with how to address potential implications and technical issues of using video-based interviews, which was approved. As educational-based research involving human subjects, who were NMC registrants, the methods of data collection were carried out in accordance with the guidelines for educational research [27] and the NMC code for nurses and midwives [28].

### 2.4. Data Collection

#### 2.4.1. Quantitative Phase Data Collection: Questionnaires

The questionnaires were created by the researchers and initially piloted in 2018 with 20 NQMs who completed their pre-registration midwifery programme from the same university in London. Based on their feedback and findings, the questionnaires were piloted again with six health sciences university staff, then twice in June–August 2019 with six NQMs who completed their pre-registration midwifery programmes from four UK universities and two SSMs from non-participating NHS Trusts, but who had similar profiles to those in this current study who met the inclusion criteria. 

Qualtrics, a password protected web-based software, was used as an adjunct to create the survey and distribute the pilot questionnaires to the small group, which allowed for testing of the link on different devices. Modifications were made as suggested to the required timing, questionnaire layout, order of questions for KASH variables as ordinal data, and the use of ‘Yes or No’ for the remaining PMH pre-registration preparation and preceptorship questions, which were nominal data. There was an additional suggestion of using a case study scenario to guide the participants in answering the set of questions on the pre-registration midwifery education and placement preparedness of the NQMs’ confidence in their KASH.

Since the participants in this current study were midwives, they were conversant to case studies and scenarios as part of their midwifery training or practice development [29]. Therefore, with reference to the main research question, the questionnaires were based on a similar case study scenario for the NQM and SSM groups, but designed to help each group of participants respond to on how well they perceived the pre-registration midwifery programme prepared the NQMs’ perinatal mental health-related KASH for their post-registration role.

Each group’s scenario was specified as follows:

- *Group 1 (NQMs): A pregnant woman accessing maternity services at your NHS Trust reports to you that she is experiencing mental distress. Has the undergraduate midwifery education and placements specific to PMH equipped you as a newly qualified midwife with sufficient confidence in your knowledge, attitude, skills, and habits to explore this woman’s mental health-related distress, discuss appropriate PMH-related care pathway, and if required, signpost the woman to relevant specialist teams or mental health services?*
- *Group 2 (SSMs): You have been working with the NQMs at your NHS Trust. Recently, a pregnant woman accessing maternity services reports to you that she is experiencing mental distress. Based on your experiences of working with the NQMs, to what extent has the undergraduate midwifery education and placements specific to PMH equipped the NQMs with sufficient confidence in their knowledge, attitude, skills, and habits to explore this woman’s mental health-related distress, discuss appropriate PMH-related care pathway, and if required, signpost the woman to relevant specialist teams or mental health services?*

The questionnaires centred on KASH variables and the participants from each group were encouraged to respond to what extent they agreed or disagreed with the KASH-specific set of questions or statements using a *5-point Likert scale* (Strongly agree, Somewhat agree, Neither agree nor disagree, Somewhat disagree, and Strongly disagree).

For the knowledge variable, the set of questions started with specific pre-existing severe mental illness (SMI) based on diagnostic criteria [30,31] which included schizophrenia, bipolar disorder and severe major depression, and other PMH conditions in light of their significant risk factors for severe maternal, foetal, and neonatal morbidity or mortality [9,32]. The knowledge variable asked whether the pre-registration specific education on PMH provided the NQMs with sufficient confidence in their knowledge of SMI and PMH conditions experienced by perinatal women or birthing people. The knowledge variable section similarly asked about the NQMs’ pre-registration preparedness on the specific PMH aspects including contributory factors to PMH issues, preventive factors, use of psychotropic medications, and the impacts of maternal mental health on the partner and children. The final sub-subsection of the knowledge variable focussed on the NQMs’ pre-registration preparedness to have sufficient knowledge of specific PMH roles, services, and suicide prevention. 

The attitude, skills, and habits variables in the questionnaires asked about the impacts of the pre-registration placements on the NQMs’ attitude towards PMH-specific roles, the NQMs’ confidence in their skills to carry out related midwifery roles or tasks, and the NQMs’ confidence to regularly discuss PMH-related questions or issues with women. 

In September 2019 to February 2020, questionnaires (*n* = 98) were distributed via the research gatekeepers from both participating NHS Trusts, based on the number meeting the inclusion and exclusion criteria. At the start of the survey, written informed consent was obtained from the participants and consent to use their data for a further research study in an anonymous manner was also sought. At the close of the survey, the participants were asked to specify their interest in participating in the focus group at their respective hospital site by providing their telephone contact details.

#### 2.4.2. Qualitative Phase Data Collection: Semi-Structured Interviews

Due to the COVID-19 pandemic, some of the survey participants who had shown interest in the focus group were not willing to participate in the semi-structured interviews. To ensure that the qualitative phase aligned with the study design section on the consolidated criteria for reporting qualitative studies (COREQ) [33], the research gatekeepers made further contact with the survey participants, and a total of twenty participants volunteered. Those who volunteered were contacted by the first author using their work email. They were then provided with participant information for the video-based online semi-structured interviews and a written consent form specifying the participants’ right to withdraw. Since the potential qualitative phase participants were frontline NHS maternity care workers with unprecedented demands due to the COVID-19 pandemic, their well-being was also considered, hence, they were all provided with links to mental health services available on the NHS to access when required.

The interview questions created by the research team were based on the main research question and the findings from the survey. The interview questions were piloted in March—April 2020 with two health sciences university staff, two NQMs, and two SSMs from non-participating NHS Trusts from the groups who previously piloted the questionnaires. Modifications were made as suggested to avoid descriptive, stereotyping, or close-ended questions, reducing the number of interview questions, but allowed more time for each participant’s discussion on KASH-related pre-registration midwifery programme preparedness experiences and using prompts only if required.

The final semi-structured interview questions were designed by the research team to ask both groups similar questions but with a slight variation to help each group of NQM and the SSM participants respond based on their specific experiences.

Some of the interview questions to each group were as follows:

- *Group 1 (NQMs):*
  ○ *As someone who has undertaken pre-registration midwifery education and placements specific to PMH, how useful was the preparation to your post-registration role?*
  ○ *In your current role, how confident are you to provide care for women or birthing people with PMH issues? Could you provide knowledge, attitude, skills, and habit specific examples?*
  ○ *What other aspects of the programme preparation do you think makes you feel less confident to provide PMH-related care and why? Could you provide knowledge, attitude, skills, and habit specific examples?*
  ○ *How do you think the aspects where you feel less confident could be improved in preparation for the PMH role? Could you provide knowledge, attitude, skills, and habit specific examples?*
  ○ *As a newly qualified midwife, are there areas or topics specific to PMH education that you feel should be covered as part of the pre-registration midwifery programme to effectively prepare NQMs for practice and why?*
  ○ *Are there placement areas or practice training specific to PMH that you feel should be part of the pre-registration midwifery programme and why?*
  ○ *Could you provide preceptorship and post-registration professional development or training specific to PMH available at your NHS Trust?*
  ○ *Are there other thoughts, ideas or recommendations to share?*
- *Group 2 (SSMs):*
  ○ *With your experience as a senior or specialist midwife working with NQMs, how well do you think the pre-registration midwifery education and placements specific to PMH prepare the NQMs for their post-registration role?*
  ○ *How confident do you think they are to provide care for women or birthing people with PMH issues? Could you provide knowledge, attitude, skills, and habit specific examples?*
  ○ *What other aspects of the programme preparation do you think makes the NQMs feel less confident to provide PMH-related care and why? Could you provide knowledge, attitude, skills, and habit specific examples based on your experience in working with NQMs?*
  ○ *How do you think the aspects where the NQMs feel less confident could be improved in preparation for the post-registration PMH role? Could you provide knowledge, attitude, skills, and habit specific examples?*
  ○ *As a senior or specialist midwife, are there areas or topics specific to PMH education you feel should be covered as part of the pre-registration midwifery programme to effectively prepare the NQMs for practice and why?*
  ○ *With your experience of working with NQMs, are there placement areas or practice training specific to PMH that you feel should be part of the pre-registration midwifery programme and why?*
  ○ *Could you provide preceptorship and post-registration professional development or training specific to PMH available to the NQMs at your NHS Trust?*
  ○ *Are there other thoughts, ideas, or recommendations specific to the NQMs’ post-registration PMH role to share?*

The interviews were conducted virtually in a quiet office or room at most of the participants’ workplaces, although some participants preferred their interviews at home. All of the participants were informed that non-participants should not be present throughout the interviews. Purposive sampling in terms of age, ethnicity, the NQMs’ training university, SSMs’ varying roles, and different hospital sites were considered for representativeness. The qualitative phase took place between May 2020 to July 2020 involving (*n* = 20) participants comprising of NQMs (*n* = 12) and SSMs (*n* = 8) with varied posts, ranging from 6 to 18 years of experience. None of the participants dropped out. All names were changed to maintain confidentiality. Interviews lasted between 17 and 47 min. The varied timing was based on the individual interview participant’s practice experiences or PMH exposure.

In light of the participants’ varying shift patterns and the overwhelming pressure of the COVID-19 pandemic, the first author carried out the interviews using guided open-ended questions that had been previously discussed and agreed with three of the authors. The first author also performed the transcription, however, to ensure trustworthiness and transparency in line with the data collection section of the COREQ standards of qualitative rigour [33], the recordings, transcription, and interpretation of the data were verified by three of the authors. The transcripts were also returned to each of the participants for comments or corrections.

No incentives were provided, but an individual message of thanks was sent to each participant, which could be used towards NMC revalidation.

### 2.5. Data Analysis

#### 2.5.1. Quantitative Phase Data Analysis

The descriptive and inferential statistical analyses were performed using Statistical Package for Social Sciences (SPSS) version 25.

For the descriptive statistics, the results of the categorical variables for both the nominal data (participants’ groups, gender, participating NHS Trusts location) and the ordinal data (the NQMs’ age group in years, both groups’ ethnicities and the SSMs’ posts) were presented as frequency and percentages (see Table 1).

For the inferential statistics, prior ‘Normality and Log Transformation’ tests were conducted to ascertain the choice of the appropriate statistical test for the comparison of responses between the NQMs and SSMs. Mann–Whitney and Wilcoxon tests were found to be appropriate and chosen to check whether there were statistical differences between the NQMs and SSMs’ ranged Likert scale responses. The statistical significance was set at *p* < 0.05 (see Table 2, Table 3, Table 4 and Table 5).

#### 2.5.2. Qualitative Phase Data Analysis

The data from the virtual interviews from 20 participants (*n* = 12 NQMs and *n* = 8 SSMs) were collated and thematically analysed using NVivo (Release 1.3 version).

Framework analysis using KASH was employed for the data analysis since the research questions focussed on the perceptions of the NQMs’ KASH, aspects perceived as useful and relevant for preparedness, and aspects perceived as not preparing the NQMs well, thus requiring improvement. Framework analysis using KASH was also amalgamated with the step-by-step approach in [33,34] involving familiarisation with the data, generating initial codes, searching for themes, reviewing themes, defining and naming themes, and writing the report. The initial coding was generated by the first author using NVivo. However, to establish the research team members’ checking, three authors also independently carried out the initial coding of the first participant’s transcript belonging to Afriyie to see whether there were any variations. The coding and the interpretation of data were cross-checked by the research team.

In alignment with the COREQ standards for qualitative research rigour [33], how to measure data saturation was discussed among the research team. Code generation from the next four participants’ transcripts were cross-checked among the research team, however, it was realised that data saturation had not been achieved in response to the research questions. It was agreed to continue with the code generation of the remaining fifteen transcripts until data saturation was reached. There was also an audit trail of the code generation and process. Following comprehensive exhaustion of the code generation from all transcripts, different codes were sorted for coherent patterns on the knowledge, attitude, skills, and habits in response to the research questions. No additional pattern or sub-theme emerged; hence data saturation had been achieved. The research team made an informed decision that the data collected from the twenty participants were comprehensive enough to generate insights on the useful and relevant aspects of the preparedness for the NQMs’ KASH and aspects requiring improvement for the NQMs’ KASH to effectively work with women with PMH needs. 

The criteria in [35] for establishing trustworthiness in qualitative research were applied at each phase of the qualitative data analysis. Credibility was promoted through prolonged engagement with the data, the research team members’ checking the transcription and coding, the return of the transcripts to each participant for verification or correction, and using direct participant quotations in the presentation of findings. The process of reflexivity was addressed by keeping reflexive journals and having regular research team meetings to discuss critical self-reflection and openness to the study, ensuring that it was data-driven, thus managing any assumptions and researcher bias. Data and method triangulation of the quantitative and qualitative phases were reviewed for shared meaning and patterns in response to the research questions.

### 2.6. Data and Method Triangulation

The overall analysis of findings reports the data and method triangulation by combining the questionnaires and semi-structured interview data collected during both phases of the study.

The quantitative data analysis for the NQMs’ knowledge, attitudes, skills, and habits (KASH) was carried out by using participants’ responses for ‘*strongly agree or agree*’ in terms of what they perceived as ‘*useful and relevant*’. In contrast, the responses of ‘*strongly disagree or disagree*’ were utilised to identify what they perceived as ‘*aspects requiring improvement’* for the NQMs’ KASH.

The reports on the KASH variables will focus on the statistically significant results, with some references to descriptive statistics where relevant (see Table 2, Table 3, Table 4 and Table 5).

## 3. Results

### 3.1. Study Participants

Out of (*n* = 98) questionnaires distributed to those who met the selection criteria, (*n* = 84) were completed, but (*n* = 2) were partially completed and excluded from analysis. Of the (*n* = 82) fully completed, (*n* = 50) were completed by the NQM participants, while (*n* = 32) were completed by the SSM participants. The 82 completed questionnaires represented an 83.6% response rate (see Table 1 showing the distributional pattern of the participants’ characteristics in the context of their gender, ethnicities, age group of the NQMs’ participants and the post held by the SSMs’ participants).

### 3.2. Research Findings on Perceptions of the NQMs’ KASH

#### 3.2.1. KASH: Knowledge Variable

The responses to the three aspects of the knowledge variable in the questionnaires and interviews are shown in Table 2.

##### The NQMs’ Confidence in Knowledge About PMH Conditions

A few of the perinatal mental health conditions and SMIs experienced by women or birthing people during pregnancy or after delivery including obsessive-compulsive disorder (OCD), fear of childbirth (FOB), and post-traumatic stress disorder (PTSD) are presented in Table 2.

##### Questionnaires

The descriptive statistics from both the NQM and SSM participants on the NQMs’ confidence in knowledge about PMH conditions are presented in Table 2, however, the findings of the ranked responses were not statistically significant (*p* > 0.05).

##### Interviews

During the interview phase, the NQMs (*n* = 8) and SSMs (*n* = 4) perceived the preparedness around anxiety and depression as ‘useful and relevant’, whereas NQMs (*n* = 5) and SSMs (*n* = 4) perceived preparing the NQMs to have sufficient confidence in the knowledge of severe mental illness conditions required improvement.

Shared perceptions from both the respective NQM and SSM groups are represented below:

“I agree …as a newly qualified midwife…that the university prepared us well for mild mental health illnesses like anxiety and depression…but when it comes to complex mental illnesses such as psychosis, I do not think we are well-prepared.” Tinashe (NQM participant).

“Mental health …issues such as anxiety and depression are common these days, they [NQMs] are probably exposed more to such conditions, than the [severe] ones like bipolar, schizophrenia, puerperia psychosis.” Afriyie (SSM participant).

### 3.3. The NQMs’ Confidence in Knowledge of Generative Mechanisms, Medications, and Impacts of PMH on the Family

#### 3.3.1. Questionnaires

The descriptive statistics implied that the NQMs were well-prepared to have sufficient confidence in their knowledge of the PMH contributory factors as well as the preventive factors, however, these ranked responses were not statistically significant. Aspects of preparedness requiring improvements were the NQMs’ confidence in the knowledge of medications to manage different PMH conditions (*p* < 0.01), and knowledge of the impacts of maternal mental health on the partner (*p* < 0.01) and children (*p* < 0.05).

#### 3.3.2. Interviews

Having sufficient knowledge of the generative mechanism is shared during the interviews by a NQM participant:

“Mental ill health is a public health issue, and it would be good to look at the wider influences of those conditions related to childbirth, the underlying pathophysiology for each condition, what could either trigger recurrence for those with the previous history or influence new onset and how these can be prevented”

Helena (NQM participant)

NQMs’ participants (*n* = 10) also confirmed that the preparedness of the NQMs to have sufficient knowledge of PMH medications required improvement. One NQM participant shared:

“Honestly, when I was qualified, I was not sure about mental health medications… I knew some and roughly [their] dosages, but …not considerations…required in pregnancy” Preeti (NQM participant).

And substantiated by SSM (*n* = 6) participants, with one stated:

“The newly qualified(s) struggle because on the midwifery pre-reg course, we do not talk much about mental health medications. Some know the common ones such as Citalopram, but when a woman says I am on clozapine, olanzapine, or any other anti-psychotics, it throws them a bit” Alex (SSM participant).

The participants explained the increasing number of women seen in practice with mental health issues and impacts on their respective families, one NQM participant shared:

“Recently on the ward, all my women either had anxiety or depression or both, think about the impacts of these common ailments on their babies, partners… so, learning should be commensurate with what is seen in practice” Gordonne (NQM participant).

Lack of supportive services for partners and fathers was also specified:

“How to support these women’s partners is not always discussed in lectures or considered even in practice and they are often the silent sufferers” Jerilyn (NQM participant).

These findings suggested knowledge deficits in these specific areas and an implication for midwifery programme curriculum review on PMH education.

### 3.4. The NQMs’ Confidence in Knowledge of PMH-Related to Midwifery and Multidisciplinary (MDT) Team Roles, Specific Services, and Suicide Prevention

#### 3.4.1. Questionnaires

Aspects perceived as ‘useful and relevant’ were the NQMs’ preparedness to have sufficient confidence in the knowledge of their PMH role, MDT role, and specific available services. These aspects were found to be statistically significant (*p* > 0.05). However, the NQMs’ preparedness on maternal suicide prevention (*p* > 0.01) was perceived as requiring improvement, with the descriptive statistics showing SSMs (62%, *n* = 20) either strongly disagreed or disagreed that the NQMs had sufficient confidence in their knowledge of maternal suicide prevention.

#### 3.4.2. Interviews

During the interviews, the NQM participants (*n* = 8) acknowledged having lectures on perinatal suicide, but not on how the midwives could help reduce maternal-related suicide. One of the NQM participants explained:

“It is daunting to have the knowledge of maternal suicide, but not the application of preventing suicides…. which… will prepare us better for future practice.” Leanne (NQM participant).

These perceptions were corroborated by the SSMs’ participants (*n* = 4), where one of them shared:

“I do not think it is our NQMs’ issue alone, it is the type of knowledge …the university and practice are passing on to them, which is sad! They are aware that women are dying violently by suicides, they can see the statistics and sincerely, most of them want to help women but with what they see in practice, the evidence of how suicide deaths can be reduced is not there as such.”

Casey (SSM participant)

In addition, during the interviews, NQM participants (*n* = 7) and SSM participants (*n* = 5) agreed that the pre-registration midwifery programme prepared the NQMs in their PMH role, emphasising the importance of making every contact count. However, some of the shared perceptions suggest that the NQMs may be lacking in the knowledge of collaborative working:

“When I qualified, we had a lady on our maternity unit who was quite poorly. She had a mental health nurse with her the whole time …changing shifts…I guess it is collaborative working. That’s just something I never thought off or came across as a student, that a pregnant or postnatal lady could be so poorly, that extra support is required”

Nikki (NQM participant)

### 3.5. The NQMs’ Confidence in the Overall Knowledge of PMH

#### 3.5.1. Questionnaires

The final question in the knowledge variable category showed a disparity in the NQMs compared with the SSMs’ responses on the NQMs’ overall confidence in their knowledge to support women with PMH needs (*p* < 0.0005).

#### 3.5.2. Interview

The differences in responses in both groups were confirmed during the interviews, with the NQM participants (*n* = 9) feeling that they had overall confidence in their knowledge to support women with PMH conditions, while the SSM participants (*n* = 6) did not feel that the NQMs had the required knowledge.

Opposing responses were re-iterated through these shared perceptions:

“The seed was planted from the first year that PMH is something that we should be considering when seeing women. So, I did not feel I was lacking in my knowledge and felt confident as a Band 5. …and to me, the university did their job by getting us to the point where we are safe to practice.”

Justilia (NQM participant)

“No, no, no, I do not think they are, I do not think the newly qualified [midwives] are well-equipped at all.” Alex (SSM participant).

In another context, one SSM participant perceived the overall NQMs’ confidence in PMH knowledge as subjective:

“The NQMs these days are more prepared ….and better equipped than the midwives who qualified years back …” Mandie (SSM participant).

### 3.6. KASH: Attitude Variable

The descriptive statistics of the NQMs and SSMs displayed in Table 3 suggest that the pre-registration midwifery placements prepared the NQMs to have a good attitude towards PMH conditions and in supporting women with PMH needs. High statistically significant results (*p* < 0.0005) were reported for the care of women’s perinatal anxiety and depression, maternal risk assessment for PMH, and the care of women living with major mental health conditions such as bipolar and schizophrenia. The NQMs’ preference to provide care for women with physical health needs as opposed to mental health needs was significant (*p* < 0.05).

#### Interviews

Similarly, during the interviews, the shared perceptions suggest that the NQMs had a good attitude towards women with PMH needs:

“Midwives are a significant part of individual woman’s maternity care story and definitely our role to support them whether they have mental health issues or not.” Preeti (NQM participant).

One participant highlighted her role as holistic, which suggests consideration and a good attitude towards the partners and family:

“Although our role is more towards women, but with experience and more knowledge, I now consider our women’s care holistically and even ask partners and family how they feel.” Nikki (NQM participant).

Some participants’ responses reflected their attitudes towards fathers, partners, and families, also acknowledging a lack of supportive services for them:

“We often forget partners, fathers, and families need support, but there is not much for them.” Tinashe (NQM participant).

“Most maternity services are women focussed. We do not have the resources for dads and partners, but we are currently promoting an app called ‘dadpad’ in my Trust. There is also a website like Dads Matter UK…” Alex (SSM participant).

One NQM participant’s shared perception showed a caring attitude, by not leaving the PMH issue to neither the midwifery nor mental health team:

“PMH is a multidisciplinary/agency problem, neither a midwifery team nor mental health team issue… When we work on women’s mental health together as a team, outcomes can be better…” Jerilyn (NQM participant).

One SSM highlighted the conflict in practice affecting the attitude towards PMH:

“As a specialist midwife, … we see hierarchy …and conflict in practice areas …affecting the attitude towards PMH including learning opportunities for the students and the new midwives.” Daveigh (SSM participant).

Different perspectives were shared by an NQM participant and an SSM participant, respectively, as contributory factors to the presumed physical care preference attitude:

“We had more sessions on other medical conditions and less on mental health, which may contribute to the reasons why most midwives feel more comfortable to care for women with non-mental health problems.” Ayan (NQM participant).

“Some of our midwives would rather care for women with other obstetric or medical issues, which is picked up by their students…., I think the students’ and the newly qualified midwives’ attitude comes down to the mentors or [practice supervisors].” Afriyie (SSM participant).

These perceptions indicate implications for pre-registration midwifery practice on PMH.

### 3.7. KASH: Skill Variable

Having sufficient confidence in the skills to carry out related PMH tasks is essential for each midwife for prompt recognition, action, and management throughout the childbirth continuum [36,37]. Table 4 presents the NQM and SSM participants’ quantitative analysis on the skill variable.

### 3.8. NQMs’ Confidence in Skills to Carry out Related PMH Tasks

#### 3.8.1. Questionnaires

The NQMs’ skills in using validated screening tools, building rapport to facilitate mental health disclosure, and recognising clients with deteriorating features were all perceived as useful and relevant aspects of pre-registration midwifery placement preparation for post-registration practice (*p* < 0.01). The overall NQMs’ confidence in their skills was reported as highly significant (*p* < 0.001) The NQMs’ skills of managing mental health emergencies (*p* < 0.05) were identified as requiring improvement, especially by the SSM participants (see Table 4).

#### 3.8.2. Interviews

Notably in this study, the NQMs used ‘Whooley questions’ as the recommended PMH screening tool at the antenatal booking in the UK, as recommended by [19] but the confidence to maintain conversations for PMH risk assessment might be lacking due to insufficient knowledge:

“I know they [NQMs] ask Whooley questions…, but …. they tend not to use the opportunity to explore what other problems women may have…You see… if they do not have enough knowledge about mental health conditions, then they will not be confident enough in how to approach PMH-related issues or ask more questions especially when the woman discloses something.”

Afriyie (SSM Participant)

“They are not confident enough in their skills to …. initiate and maintain mental health conservations with women. They sometimes feel uncomfortable when a woman is crying.” Casey (SSM participant).

These perceptions were substantiated by the NQMs including not using their initiatives to pick up non-verbal cues:

“Effective use of something as presumed simple as the Whooley questions would be good as part of practice preparation. I know most [students and NQMs] are not comfortable to ask women, how is your mood? Most rarely use their initiatives …. even when the lady sat there looking terrible.” Gordonne (NQM participant).

“Again, it is difficult, when it comes to practice, for example, in terms of how you go about supporting someone …practically… are not usually covered at the university…such as… starting the conservation with the woman, or what to do if a woman starts crying, showing signs of needing help.” Chazielle (NQM participant).

Dealing with mental health emergencies was iterated as one of the areas identified by the NQM and SSM participants where additional skill was required:

“Midwives generally lack skill in dealing with mental health emergencies or crises, because it is not part of our training.” Helena (NQM participant).

“I think incorporating mental health emergencies into managing obstetric emergencies…mandatory training…both at the university and in practice would be fantastic for students to develop their mental health skills before they qualify.” Mariliki (SSM participant).

The shared perceptions from the NQM and SSM participants indicated the NQM individuality regarding the confidence in carrying out PMH tasks:

When I qualify… I felt… prepared and confident in terms of everything really…, …. We learn a lot in three years and …the lecturers teach us …vast amount…. How do you physically squeeze more into the three years?” Justilia (NQM participant).

“Although training is important, but it is also an individual thing for most of our preceptees in terms of how comfortable, confident, and competent they feel in carrying out mental health-related tasks …and caring for these women.” Temi (SSM participant).

### 3.9. KASH: Habits Variable

Professional ‘habits’ as part of the KASH educational framework [25] relate to promoting excellence in care through PMH-related discussions with women, which the NQMs should carry out on a regular basis (see Table 5 for the quantitative analysis of the ‘habits’ variable).

### 3.10. NQMs’ Confidence to Regularly Discuss PMH-Related Questions or Issues with Women

#### 3.10.1. Questionnaires

Aspects of preparedness perceived as useful and relevant with statistically significant results were the NQMs’ having sufficient confidence to regularly discuss the maintenance of mental health and well-being at the antenatal booking (*p* < 0.05) and making prompt referrals (*p* < 0.01).

Aspects of pre-registration midwifery placements requiring improvements, where over 50% of the SSMs either strongly disagreed or disagreed were the NQMs with sufficient confidence to regularly discuss suicidal thoughts (*p* < 0.01) and issues of self-harm with women (*p* < 0.05). A discrepancy in both the responses of the NQMs and SSMs was noted in the NQMs’ confidence to regularly debrief women who had pregnancy loss or neonatal death (*p* < 0.05).

#### 3.10.2. Interviews

The lived experiences shared suggest the importance of having a habit of asking women questions to prevent missing important previous severe mental health history:

“I booked a lady just before I qualified, who did not make any disclosure, but when I went through her records online, I discovered that she has had repeated A&E admissions 6 months prior to her booking, and they were all suicide attempts.” Helena (NQM participant).

“One of my ladies with known severe mental health problems…and previous crisis… had her …booking appointment … at the hospital but did not disclose the severity of her condition. She later explained to me that the…booking midwife …was rushing and did not ask her the details.” Williamme (SSM participant).

Lack of time may also contribute to the inability to routinely ask about their mental health needs, contributing to some slipping through the net:

“It is difficult, because when you are newly qualified, you are trying to find your feet, especially on delivery suite or on a busy postnatal ward. I feel like you do not really spend enough time with the women to regularly talk to them about their feelings …. You do not see that side when you are training. Now I understand more why some women slip through the net which should not be.”

Johannah (NQM participant)

Inability to ask challenging PMH history was attributed to the lack of pre-registration midwifery preparedness:

“No, …we are …not really prepared on how to…routinely… ask questions…relating to PMH crisis or anything that may… indicate … high risk of suicide.” Leanne (NQM participant).

The NQM participants (*n* = 6) perceived that they had sufficient confidence to make referrals to the appropriate multi-agency services:

“When I started as a newly qualified… I felt confident enough to make a referral to the right [mental health] services. I can always ask if unsure.” Tinashe (NQM participant).

Conversely, the SSM participants (*n* = 4) did not perceive that the NQMs had confidence due to their lack of knowledge or skills, which likely explained the opposing views:

“Referrals and where to signpost to? In my opinion, they do not have that confidence… It is all linked with their skills and ability to effectively… risk assess. Sometimes our lovely newly qualified midwives will refer women with anxiety but not women with bipolar.” Alex (SSM participant).

“Making …regular… PMH referrals can be tricky for most of the preceptees in terms of what they know …., and where they trained…. Generally, in terms of their confidence in how to refer and where to refer, they are not there yet.” Temi (SSM participant).

From the qualitative findings, regular support from senior specialist midwives generally was mentioned by some NQMs as useful in facilitating the incorporation of PMH care habit into their regular practice at every antenatal or postnatal contact:

“I did not have the confidence to start any conversations on PMH with women when I started …as a newly qualified… but our specialist PMH midwife is so good and approachable. She goes through the resources I could use to discuss mental health issues with women, and now I have more confidence to use resources regularly.”

Ayan (NQM participant)

One SSM participant suggested that the NQMs attending the debriefing service and fear of childbirth pathway clinic could help them to learn how to talk to women:

“The NQMs do struggle talking with women who have experienced some form of psychological trauma, …or previous losses… In my Trust, we run a debriefing service, and another clinic for women with tocophobia. They can come and sit with us to hear how women are debriefed and even see how we use fear of childbirth pathway…depending on whether it is…primary or secondary.”

Alex (SSM participant)

## 4. Discussion

Pre-registration midwifery PMH education and placement preparedness have an impact on the NQMs’ relevant KASH and their level of confidence in their knowledge, attitude to PMH roles, skills, and habits to effectively support women or birthing people and families with PMH needs as part of their post-registration practice. This study’s theoretical framework and the interconnections of social constructivism, critical realism, and pragmatism explaining the NQMs’ KASH and the impact on PMH role preparedness addressing the research question are presented in Figure 1 [26]. The pragmatism paradigm provided the ontological, epistemological, methodological, and axiological positions connected to this study, which explains the multifaceted perspectives and perceptions around the NQMs’ pre-registration midwifery PMH education and placement preparedness for post-registration practice. This article specifically focussed on the perceptions of usefulness, relevance of the NQMs’ KASH, and aspects required for the NQMs’ KASH. The perceptions of barriers and strategies to PMH education and placements (see Figure 1) are implications for future research.

The study participants in both the NQM and SSM groups were female; hence, males were not represented. This under-representation might be due to midwifery focusing on women-centred care and childbirth, which likely accounts for professional attraction to females [38,39]. Although most of the study participants were from a White British ethnic background, diverse ethnicities from White Irish, or European; Mixed heritage, Black British, African or Caribbean, and Asian British or Asian backgrounds were also represented.

As shown in Table 1 for the NQMs’ age group, most of the participants (*n* = 22) 44% were between 21 years and under, and 22–25 years old, while (*n* = 12) 24% were between 26 and 30 years. Apart from PMH being part of the NQMs’ midwifery role, there is a possibility of having shared interest in PMH due to the awareness of mental illness realities [40] or because of changes to the context in which the midwives work in the last decade [41,42]. There has been an increase PMH needs because of the complexities of maternity population profiles, growing inequalities, and the wider social determinants of health and rapidly changing healthcare needs, along with advancing technological developments and the societal expectations of the profession [12,41,43].

The 50 NQM participants completed their undergraduate midwifery education from ten different UK universities between January 2018 and December 2019. Nevertheless, regardless of the universities attended in the UK, all of the NQM participants must have met the same pre-2019 midwifery standards as required at the point of registration to the midwifery part of the register [18].

In this current study, shared perceptions from both the NQM and the SSM participants explained how well the NQMs were prepared in certain aspects to support women with PMH needs and the aspects of preparedness requiring improvement. Notably, there was a reported disparity between the strongly agreed or agreed responses of the NQMs and SSMs that the pre-registration midwifery programme prepared the NQMs to have an overall knowledge of PMH to support women (NQMs 80% versus SSMs 43.7%; *p* < 0.0005). However, nearly the same percentage of the SSM respondents, 40.6%, either strongly disagreed or disagreed. This finding probably highlights the NQMs’ self-confidence in their overall knowledge to support women, while nearly 60% of the SSMs who worked with the NQMs disputed that they had the knowledge. A contradictory result indeed, but highly significant (*p* < 0.0005). As of the time of this current study, it appears that there are limited reports on the comparison between the NQMs and SSMs’ perceptions around the NQMs’ knowledge of PMH. Therefore, one could conclude that there is a potential gap between the NQMs and SSMs’ perceptions in this area of preparedness, which requires further clarification. Future research with a larger sample size of NQMs and SSMs from varying maternity units may provide more insights into the different perceptions of this aspect of pre-registration midwifery programme preparedness on the NQMs’ PMH role.

This current study confirmed findings from previous studies [24,44,45,46,47] that some midwives find it challenging to have sufficient knowledge of psychotropic medications, (*p* < 0.01) severe PMH conditions, and related risk assessments. Pre-existing mental health or PMH issues are significant risk factors for severe maternal, foetal, and neonatal morbidity or mortality [9,32]. A lack of pre-registration midwifery programme preparation of the NQMs to have sufficient knowledge of the major or severe mental illness, which includes schizophrenia, bipolar disorder, and severe major depression [30,31], was particularly specified during the semi-structured interviews by both groups in the current study’s participants. Notably in the MBRRACE-UK reports [10,11,20], most women who died by suicide or substance misuse had established psychiatric varying diagnoses including recurrent depressive episode, bipolar affective disorder, schizophrenia or schizoaffective disorder, and definite or probable psychosis at the time of their death.

Ref. [10] also reported that 75% of the women who died by using substance misuse had a mental health diagnosis, while [48,49,50] reported multiple complex adversities including domestic abuse among the maternity population, leading to internal struggles, drug and alcohol misuse, and self-harm in some cases. Most of these women had contact with midwives and maternity care professionals, and the PMH issues were either not promptly identified or ineffectively managed, resulting in poor perinatal outcomes [51]. These reports also signify an educational concern for the midwives and multidisciplinary teams, emphasising the importance of evidence-based multidisciplinary [10,11,20,49,51].

It may be useful to ensure that developers and educators of the pre-registration midwifery curriculum review how best the NQMs can be prepared in relation to their PMH role. The process of how midwifery programme curriculum developers can review the PMH processes of the university and placements is one of the recommendations for future research. These processes specifically address the deficits of the NQMs’ knowledge, skills, and habits around perinatal health conditions, PMH-related aspects including psychotropic medications, available supportive services for PMH needs, reducing maternal-related suicide, skills for managing mental health emergencies, and regular habits to address PMH issues at every perinatal contact.

As reported in this study, specific preceptorship exposure such as allocated time during the preceptorship programme with the specialist PMH team, regular support from the senior midwives, and post-registration PMH simulated training as part of the mandatory yearly update could contribute to increasing the NQMs’ confidence in PMH knowledge, maintaining a good attitude, improving skills in carrying out related PMH tasks, and having a regular discussion of PMH issues. This approach indicates an implication for the preceptorship programme.

The knowledge of the NQMs on the impact of maternal mental health on partners (*p* < 0.01) and children (*p* < 0.05) required improvement. Partners and fathers are still silent sufferers, as pointed out by some of the NQM respondents in this study, with 40% of the qualitative phase’s participants suggesting the need for inclusive services and resources on maternity healthcare for fathers, partners, and the entire household, and that training on improving paternal mental health should be part of the preparedness on pre-registration education and practice. Recent evidence indicates that supporting paternal mental health is gradually gaining the attention of the policymakers, healthcare service commissioners, and health professionals [52,53,54]. Internationally, [55] in their quasi-experimental design study in Germany, also reported that the highly stressed fathers were not recognised by the midwives. Similarly, the midwives participating in a multi-method approach study in Australia conveyed the importance of engaging fathers as part of their PMH role [56]; recognising the significance of training needs for midwifery practitioners was also reported [56]. This finding has potential implications for maternity healthcare services, policymakers, pre-registration midwifery education, placements, and post-registration professional development. How the midwifery programme curriculum developers can address the NQMs’ knowledge deficits on the impacts of the maternal mental health on partners, fathers, and children including available supportive services and family centred inclusive resources for partners, fathers, and families are recommendations for future research.

This current study also reports the sufficient preparation of the NQMs with confidence in their skills to build rapport to facilitate the disclosure of mental health issues and recognise deteriorating PMH (*p* < 0.01). It should, however, be noted that women being unenthusiastic about engaging with maternity or overall healthcare could also be an indicator of worsening mental health issues, which may be triggered by multiple adversities, traumatic obstetric-related experiences, poor neonatal outcomes or perinatal losses at any stage in pregnancy, ongoing safeguarding concerns, and social services proceedings [50,57,58] as well as severe PMH morbidity [59,60].

Effective pre-registration midwifery preparation at both university and during placements should recognise the importance of MDT collaborative working with women or birthing people who are vulnerable to aid prompt provision of individualised care including swift signposting and shared follow-up [14,61].

Again, as seen in the perceptions of knowledge section, the assessment of the NQMs’ confidence in using their skills to support women identified a disparity between the NQMs (76%) and SSMs (40.6%) in the ranked Likert scale in the strongly agreed or agreed responses over the NQMs having overall confidence in their skills (*p* = 0.001). It is not entirely clear why the rating scale between the two groups were vastly different in certain areas. There is a possibility that the experiences of the SSMs supporting women living with PMH compared to their observations when working with the NQMs might have contributed to their lower agreed or agreed responses. Therefore, it is worth considering whether the SSMs were biased in their responses over the NQMs’ confidence in their PMH-related skills or if the NQMs were over-rating themselves.

In this study, it appears that the more confident the NQMs were in their PMH knowledge and using PMH-related skills, the higher the possibility of regular discussions on PMH-specific issues such as self-harm, suicidal thoughts, debriefing women following perinatal losses, and making use of relevant resources. These findings suggest gaps in their pre-registration preparedness, and thus have implications for midwifery education and practice developments. The NQM and SSM participants provided KASH sustenance suggestions including pre-registration healthcare students’ interprofessional learning and PMH case management through adaptable simulation, midwifery-specific practical training using anonymised scenarios, PMH placement exposure, elective placements, and preceptorship PMH learning.

Some NQMs or future midwives may find providing care for women with PMH needs emotionally draining or may trigger underlying issues or their own personal experiences. Therefore, an emphasis on an awareness of self-vulnerability, provision of emotional resilience training and interprofessional learning in the pre-registration midwifery programme specific to PMH education and placements is vital to facilitate self-care and build an emotionally resilient maternity workforce.

### Strengths and Limitations

This current sequential explanatory mixed methods design has provided insights into how and why the appropriate KASH should take the centre stage for the effective pre-registration midwifery PMH education, practice, and preceptorship preparedness of NQMs. Previous studies [21,22,62,63,64,65] have explored the midwives’ knowledge, attitudes, and skills of PMH; however, this study contributes to the body of knowledge through the unique interconnections of social constructivism, critical realism model, pragmatism, and fourfold KASH framework to explore the NQMs’ preparedness for the PMH role (see Figure 1 [26]). It also appears that there are limited studies on the comparison between the NQM and SSM independent groups’ perceptions around the NQMs’ KASH of PMH education and practice preparedness.

However, the study was limited to the 82 NQM and SSM participants from two NHS Trusts in South-East London and North Kent, with the NQMs representing ten UK universities. Therefore, the findings of this study may not be generalised to other parts of the UK and other countries with different maternity population profiles, PMH wider influences, or NQM and SSM workforce. However, the findings could be transferable to studies conducted in similar contexts. Similarly, future research with national and international NQM and SSM participants working with women or birthing people from varying population backgrounds and wider social determinants may provide clearer insights into the midwives’ perceived KASH and dynamic professional development that is practical, universally inclusive, and culturally acceptable. The NQM participants were from ten different universities with varying pre-registration midwifery PMH education and placement experiences, and the differences between PMH curriculum and placements of varying study backgrounds could not be controlled on their perceptions, which could be a limitation of this study.

The COVID-19 pandemic was an unforeseeable limitation that had an impact on the second phase of data collection, contributing to changes from the planned focus group to video-based online semi-structured interviews using the Microsoft Teams platform. It is therefore acknowledged that face-to-face group discussion could have provided opportunities for social interactions and possibly different results. Although the qualitative data from 20 respondents were rich to complement the quantitative data, future research using focus groups may possibly clarify some of the aspects identified.

## 5. Conclusions

There were useful and relevant aspects identified through this study to ascertain how the pre-registration midwifery education and placements prepared the NQMs’ KASH to a certain extent for their post-registration PMH role, but there were perceived aspects requiring KASH improvement. To facilitate the NQMs’ sustainable confidence in their PMH-related KASH, perceived areas for improvement have implications for pre-registration midwifery-specific PMH education and placement development, specific post-registration PMH education, planning and policy developments, and preceptorship programme development to consolidate PMH pre-registration education and practice. Future research will identify specific barriers to the NQMs’ PMH role preparedness and the strategies to facilitate effective PMH role preparedness.

## Figures and Tables

**Table 1:** The participants’ background variables.

Background Variables	Frequency (of 82)	Percentages (%)
**Participants:**		
NQMs	50	61
SSMs	32	39
**Gender:**		
Male	0	0
Female	82	100
Prefer not to say	0	0
**Ethnicities: NQMs (*n* = 50)**		
White British/English	35	70
White Irish	2	4
White European/Other	3	6
Mixed (White/Caribbean)	1	2
British Non-Specified	2	4
Black British	2	4
Black African	2	4
Caribbean	1	2
Asian/Asian British	2	4
I prefer not to say	0	0
**Ethnicities: SSMs (*n* = 32)**		
White British/English	22	69
White European/Other	3	10
Mixed (White/Caribbean)	1	3
British Non-Specified	2	6
Black British	1	3
Black African	2	6
Caribbean	0	0
Asian/Asian British	0	0
I prefer not to say	1	3
**Age group in years: NQMs (*n* = 50)**		
21 and under	4	8
22–25	18	36
26–30	12	24
31–35	8	16
36–40	3	6
41–45	2	4
46–50	3	6
I prefer not to say	0	0
**Posts held by the SSMs (*n* = 32)**		
Senior Midwife—Band 7	16	50
Clinical Practice Facilitator (CPF)	5	16
Perinatal Mental Health Midwife	3	10
Preceptorship Lead Midwife	2	6
Midwife—Band 6 in a specialist post	2	6
Practice Development Midwife	1	3
Matron	1	3
Other specialist midwife	1	3
I prefer not to say	1	3

Abbreviations: NQMs—newly qualified midwives; SSMs—senior specialist midwives.

**Table 2:** The findings of the NQMs and the SSMs on the NQM’s confidence in the PMH-related KASH—Knowledge variable.

KASH Variables Knowledge of							
PMH Conditions:	NQMs Strongly Agree/Agree *n* = 50 (%)	NQMs Strongly Disagree/Disagree	Neutral	SSMs Strongly Agree/Agree *n* = 32 (%)	SSMs Strongly Disagree/Disagree	Neutral	Mann–Whitney U *p*-Value Asymp.Sig. (2-Tailed)
Anxiety	36 (72%)	8 (16%)	6 (12%)	20 (62.5%)	8 (25%)	4 (12.5%)	0.158
Depression	39 (78%)	8 (16%)	3 (6%)	24 (75%)	6 (19.4%)	2 (6.3%)	0.212
Psychosis	27 (54%)	15 (30%)	8 (16%)	13 (40.6%)	13 (40.6%)	6 (18.8%)	0.138
Bipolar	17 (34%)	23 (46%)	10 (20%)	10 (31.3%)	19 (59.4%)	3 (9.4%)	0.493
Schizophrenia	12 (24%)	29 (58%)	9 (18%)	5 (15.6%)	21 (65.7)	6 (18.8%)	0.629
OCD	17 (34%)	24 (48%)	9 (18%)	7 (21.9%)	18 (56.2%)	7 (21.9%)	0.361
FOB	34 (68%)	9 (18%)	7 (14%)	23 (71.9%)	8 (25.1%)	1 (3.1%)	0.239
PTSD	30 (60%)	11 (22%)	9 (18%)	15 (46.9%)	14 (43.8%)	3 (9.4%)	0.061
***Specific PMH-related aspects:***							0.118
Contributory factors	42 (84%)	5 (10%)	3 (6%)	26 (81.3%)	2 (6.3%)	4 (12.5)	
Preventive factors	36 (72%)	8 (16%)	6 (12%)	18 (56.2%)	7 (21.9%)	7 (21.9%)	0.057
Medications	19 (38%)	21 (42%)	10 (20%)	4 (12.5%)	23 (71.9%)	5 (15.6%)	0.004 **
Impact on partner	26 (52%)	15 (30%)	9 (18%)	10 (31.3%)	20 (62.5%)	2 (6.3%)	0.009 **
Impact on children	24 (48%)	15 (30%)	11 (22%)	12 (37.5%)	16 (50%)	4 (12.5%)	0.048 *
***Specific roles, services, and suicide prevention:***							0.018 *
PMH midwives’ role	38 (76%)	9 (18%)	3 (6%)	19 (59.4%)	10 (31.2%)	3 (9.4%)	
MDT role	40 (80%)	9 (18%)	1 (2%)	22 (68.8%)	8 (25.1%)	2 (6.3%)	0.013 *
Available services	36 (72%)	9 (18%)	5 (10%)	19 (59.4%)	10 (31.2%)	3 (9.4%)	0.044 *
Suicide prevention	24 (48%)	16 (32%)	10 (20%)	6 (18.8%)	20 (62.5%)	6 (18.8%)	0.003 **
Overall knowledge confidence	40 (80%)	5 (10%)	5 (10%)	14 (43.7%)	13 (40.6%)	5 (15.6%)	0.000 ***

Abbreviations: KASH: knowledge, attitude, skills, and habits; PMH: perinatal mental health; OCD: obsessive-compulsive disorder; FOB: fear of childbirth; PTSD: post-traumatic stress disorder; MDT: multidisciplinary team; MH: mental health; AN: antenatal; PN: postnatal; NND: neonatal death. The pre-registration midwifery education provided the NQMs with sufficient confidence in their knowledge to recognise women with the following PMH conditions, PMH-related aspects, specific roles, available services, and suicide prevention. Statistical significance was set at *p* < 0.05 and in bold. *p* < 0.05 *, *p* < 0.01 **, *p* < 0.001 ***, *p* = 0.000 (*p* < 0.0005 ***). *p* > 0.05 = NS: non-significant, and not in bold.

**Table 3:** Findings of the NQMs and the SSMs on the NQM’s confidence in the PMH-related KASH—Attitude variable.

KASH Variables: Attitude							
*NQMs’ Attitude Towards:*	NQMs Strongly Agree/Agree *n* = 50 (%)	NQMs Strongly Disagree/ Disagree	Neutral	SSMs Strongly Agree/ Agree *n* = 32 (%)	SSMs Strongly Disagree/ Disagree	Neutral	Mann–Whitney U *p*-Value Asymp.Sig. (2-Tailed)
Maternal PMH assessment	46 (92%)	3 (6%)	1 (2%)	27 (84.4%)	4 (12.5%)	1 (3.1%)	0.000 ***
Women living with anxiety and depression	48 (96%)	1 (2%)	1 (2%)	25 (78.1%)	3 (9.4%)	4 (12.5%)	0.000 ***
Women living with major mental health conditions	45 (90%)	3 (6%)	2 (4%)	16 (50.1%)	8 (25%)	8 (25%)	0.000 ***
PMH women’s management as the responsibility of MH team	39 (78%)	5 (10%)	6 (12%)	24 (75%)	4 (12.5%)	4 (12.5%)	0.204
Physical health needs as opposed to MH needs	15 (30%)	13 (26%)	22 (44%)	21 (65.6%)	7 (21.9%)	4 (12.5%)	0.025 *

Abbreviations: KASH: knowledge, attitude, skills, and habits; NQMs: newly qualified midwives; SSMs: senior specialist midwives; PMH: perinatal mental health; MH: mental health. NQMs’ attitude to their PMH midwifery-specific role. Statistical significance was set at *p* < 0.05 and in bold. *p* < 0.05 *, *p* < 0.001 ***, *p* = 0.000 (*p* < 0.0005 ***). *p* > 0.05 = NS: non-significant, and not in bold.

**Table 4:** Findings of the NQMs and the SSMs on the NQM’s confidence in the PMH-related KASH—Skills variable.

KASH Variables: Skills							
NQMs’ Have Confidence in Their Skills to:	NQMs Strongly Agree/Agree *n* = 50 (%)	NQMs Strongly Disagree/ Disagree	Neutral	SSMs Strongly Agree/Agree *n* = 32 (%)	SSMs Strongly Disagree/ Disagree	Neutral	Mann–Whitney U *p*-Value Asymp.Sig. (2-Tailed)
Using validated tools for PMH assessment	37 (74%)	6 (12%)	7 (14%)	15 (46.9%)	12 (37.6%)	5 (15.6%)	0.005 **
Building rapport to facilitate disclosure.	42 (84%)	5 (10%)	3 (6%)	21 (65.6%)	7 (21.9%)	4 (12.5%)	0.004 **
Recognise deteriorating PMH.	41 (82%)	6 (12%)	3 (6%)	18 (56.3%)	9 (28.2%)	5 (15.6%)	0.007 **
Managing MH emergencies.	23 (46%)	18 (36%)	9 (18%)	7 (21.9%)	17 (53.2%)	8 (25%)	0.038 *
Overall skills confidence	38 (76%)	4 (8%)	8 (16%)	13 (40.6%)	11 (34.4%)	8 (25%)	0.001 ***

Abbreviations: KASH: knowledge, attitude, skills, and habits; NQMs: newly qualified midwives; SSMs: senior specialist midwives; PMH: perinatal mental health. MH: mental health. NQMs’ confidence in their skills to carry out related PMH midwifery roles. Statistical significance was set at *p* < 0.05 and in bold. *p* < 0.05 *, *p* < 0.01 **, *p* < 0.001 ***, *p* = 0.000 (*p* < 0.0005 ***). *p* > 0.05 = NS: Non-significant, and not in bold.

**Table 5:** Findings of the NQMs and the SSMs on the NQM’s confidence in the PMH-related KASH—Habits variable.

KASH Variables: Habits							
NQMs’ Confidence to Regularly Discuss:	NQMs Strongly Agree/Agree *n* = 50 (%)	NQMs Strongly Disagree/ Disagree	Neutral	SSMs Strongly Agree/Agree *n* = 32 (%)	SSMs Strongly Disagree/ Disagree.	Neutral	Mann–Whitney U *p*-Value Asymp.Sig. (2-Tailed)
Maintenance of MH and well-being at booking.	39 (78%)	8 (16%)	3 (6%)	22 (68.8%)	6 (18.7%)	4 (12.5%)	0.033
Maintenance of MH and well-being at every AN contact.	33 (66%)	12 (24%)	5 (10%)	20 (62.5%)	9 (28.2%)	3 (9.4%)	0.156
Maintenance of MH and well-being at every PN contact	38 (76%)	9 (18%)	3 (6%)	28 (87.6%)	4 (12.5%)	0 (0%)	0.097
Suicidal thoughts with women	27 (54%)	17 (34%)	6 (12%)	9 (28.1%)	18 (56.3%)	5 (15.6%)	0.009 **
Issues of self-harm with women	24 (48%)	20 (40%)	6 (12%)	8 (24%)	19 (59.4%)	5 (15.6%)	0.043 *
Use leaflets/other resources to discuss MH issues with women.	35 (70%)	8 (16%)	7 (14%)	23 (71.9)	7 (21.9%)	2 (6.3%)	0.215
Make prompt referrals for women at risk or living with MH.	43 (86%)	4 (8%)	3 (6%)	21 (65.7%)	6 (18.7%)	5 (15.6%)	0.005 *
Debrief women with unexpected outcomes—pregnancy loss, NND	28 (56%)	13 (26%)	9 (18%)	11 (34.4%)	14 (43.8%)	7 (21.9%)	0.022 *

Abbreviations: KASH: knowledge, attitude, skills, and habits; PMH: perinatal mental health; MH: mental health; AN: antenatal; PN: postnatal; NND: neonatal death. NQMs’ confidence to regularly discuss PMH-related questions or issues with women. (Statistical significance is set at *p* < 0.05 and in bold. *p* < 0.05 *, *p* < 0.01 **. *p* > 0.05 = NS: non-significant, and not in bold.

## Data Availability

Quantitative and qualitative data are contained within this article.

## References

[1] Howard L.M., Khalifeh H. (2020). Perinatal mental health: A review of progress and challenges. World Psychiatry.

[2] Bauer A., Knapp M., Parsonage M. (2016). Lifetime costs of perinatal anxiety and depression. J. Affect. Disord..

[3] Jones D., Hazelton M., Ebert L. (2015). Perinatal mental health and men. Aust. Nurs. Midwifery J..

[4] Gibson J., Gray R., Martin C. (2012). Epidemiology of maternal mental health disorders. Perinatal Mental Health. A Clinical Guide.

[5] Anderson F.M., Hatch S.L., Comacchio C., Howard L.M. (2017). Prevalence and risk of mental disorders in the perinatal period among migrant women: A systematic review and meta-analysis. Arch. Womens Ment. Health.

[6] Howard L.M., Piot P., Stein A. (2014). No health without perinatal mental health. Lancet.

[7] Underwood L., Waldie K.E., D’Souza S., Peterson E.R., Morton S.M.B. (2017). A Longitudinal Study of Pre-pregnancy and Pregnancy Risk Factors Associated with Antenatal and Postnatal Symptoms of Depression: Evidence from Growing Up in New Zealand. Matern. Child Health J..

[8] Ford E., Shakespeare J., Elias F., Ayers S. (2017). Recognition and management of perinatal depression and anxiety by general practitioners: A systematic review. Fam. Pract..

[9] Jones I., Chandra P.S., Dazzan P., Howard L.M. (2014). Bipolar disorder, affective psychosis, and schizophrenia in pregnancy and the post-partum period. Lancet.

[10] Cantwell R., Cairns A., Bunch K., Knight M., Knight M., Bunch K., Tuffnell D., Patel R., Shakespeare J., Kotnis R., Kenyon S., Kurinczuk J.J., MBRRACE-UK Mental Health Chapter-Writing Group (2021). Improving mental health care and care for women with multiple adversity. Saving Lives, Improving Mothers’ Care—Lessons Learned to Inform Maternity Care from the UK and Ireland Confidential Enquiries into Maternal Deaths and Morbidity 2017–2019.

[11] Cantwell R., Youd E., Knight M., Maternal Report 2018, Knight M., Bunch K., Tuffnell D., Jayakody H., Shakespeare J., Kotnis R., Kenyon S., Kurinczuk J.J. (2018). Messages for mental health. Saving Lives, Improving Mothers’ Care—Lessons Learned to Inform Maternity Care from the UK.

[12] Jones G.L., Mitchell C.A., Hirst J.E., Anumba D.O.C. (2022). Understanding the relationship between social determinants of health and maternal mortality. BJOG Int. J. Obstet. Gynaecol..

[13] France-Williams A. (2017). New Report Published on Maternal Deaths and Morbidity.

[14] Renfrew M.J., McFadden A., Bastos M.H., Campbell J., Channon A.A., Cheung N.F., Silva D.R., Downe S., Kennedy H.P., McCormick F. (2014). Midwifery and quality care: Findings from a new evidence-informed framework for maternal and newborn care. Lancet.

[15] International Confederation of Midwives (ICM) (2010). Global Standards for Midwifery Education (2010) Amended 2013. https://www.internationalmidwives.org/wp-content/uploads/global-standards-for-midwifery-education_2021_en.pdf.

[16] International Confederation of Midwives (ICM) (2019). Essential Competencies for Midwifery Practice.

[17] Nursing and Midwifery Council (NMC) (2019). Standards of Proficiency for Midwives.

[18] Nursing and Midwifery Council (NMC) (2020). Pre-2019 Standards.

[19] NICE (2014). Antenatal and Postnatal Mental Health: Clinical Management and Service Guidance.

[20] Cantwell R., Knight M., Oates M., Shakespeare J., Knight M., Tuffnell D., Kenyon S., Shakespeare J., Gray R., Kurinczuk J.J. (2015). Lessons on maternal mental health. Saving Lives, Improving Mothers’ Care—Surveillance of Maternal Deaths in the UK 2011-13 and Lessons Learned to Inform Maternity Care from the UK and Ireland Confidential Enquiries into Maternal Deaths and Morbidity 2009–2013.

[21] Savory N.A., Sanders J., Hannigan B. (2022). Midwives’ experiences of supporting women’s mental health: A mixed-method study. Midwifery.

[22] Noonan M., Jomeen J., Galvin R., Doody O. (2018). Survey of midwives’ perinatal mental health knowledge, confidence, attitudes and learning needs. Women Birth.

[23] Higgins A., Downes C., Monahan M., Gill A., Lamb S.A., Carroll M. (2018). Barriers to midwives and nurses addressing mental health issues with women during the perinatal period: The Mind Mothers study. J. Clin. Nurs..

[24] McCauley K., Elsom S., Muir-Cochrane E., Lyneham J. (2011). Midwives and assessment of perinatal mental health. J. Psychiatr. Ment. Health Nurs..

[25] Griffith A., Burns M., Owen N. (2014). Outstanding Teaching: Teaching Backwards.

[26] Onilude Y. (2021). The Interconnections of Social Constructivism, Critical Realism and Pragmatism to Explain the NQMs’ KASH and the Impact on PMH Role Preparedness. Ph.D. Thesis.

[27] British Educational Research Association (BERA) (2018). Ethical Guidelines for Educational Research.

[28] Nursing and Midwifery Council (NMC) (2018). The Code: Professional Standards of Practice and Behaviour for Nurses, Midwives and Nursing Associates.

[29] Harvey M., Land L., Seaman J. (2017). Research Methods for Nurses and Midwives: Theory and Practice.

[30] American Psychiatric Association (2022). Diagnostic and Statistical Manual of Mental Disorders, Text Revision (DSM-5-TR), 5th ed.

[31] American Psychiatric Association (2013). Diagnostic and Statistical Manual of Mental Disorders: DSM-5.

[32] Anderson M. (2022). Midwifery Essentials. Perinatal Mental Health.

[33] Tong A., Sainsbury P., Craig J. (2007). Consolidated criteria for reporting qualitative research (COREQ): A 32-item checklist for interviews and focus groups. Int. J. Qual. Health Care..

[34] Braun V., Clarke V. (2006). Using thematic analysis in psychology. Qual. Res. Psychol..

[35] Lincoln Y.S., Guba E.G. (1985). Establishing Trustworthiness. Naturalistic Inquiry.

[36] Royal College of Midwives (RCM) (2015). Caring for Women with Mental Health Problems Standards and Competency Framework for Specialist Maternal Mental Health Midwives.

[37] Wadephul F., Jarrett P.M., Jomeen J., Martin C.R. (2018). A mixed methods review to develop and confirm a framework for assessing midwifery practice in perinatal mental health. J. Adv. Nurs..

[38] Berkery E., Tiernan S., Morley M. (2014). The relationship between gender role stereotypes and requisite managerial characteristics: The case of nursing and midwifery professionals. J. Nurs. Manag..

[39] Kouta C., Kaite C.P. (2011). Gender Discrimination and Nursing: A Literature Review. J. Prof. Nurs..

[40] Gunnell D., Kidger J., Elvidge H. (2018). Adolescent mental health in crisis. BMJ.

[41] Nursing and Midwifery Council (NMC) (2019). NMC to Reshape Midwifery Care for Next Generation.

[42] Nursing and Midwifery Council (NMC) (2019). NMC Sets Out Proposals to Transform the Future of Midwifery Care Across the UK.

[43] Ban L., Gibson J.E., West J., Fiaschi L., Oates M.R., Tata L.J. (2012). Impact of socioeconomic deprivation on maternal perinatal mental illnesses presenting to UK general practice. Br. J. Gen. Pract..

[44] de Vries N.E., Stramrood C.A.I., Sligter L.M., Sluijs A.M., van Pampus M.G. (2020). Midwives’ practices and knowledge about fear of childbirth and postpartum posttraumatic stress disorder. Women Birth.

[45] Molenaar N.M., Brouwer M.E., Duvekot J.J., Burger H., Knijff E.M., Hoogendijk W.J., Bockting C.L., de Wolf G.S., Lambregtse-van den Berg M.P. (2018). Antidepressants during pregnancy: Guideline adherence and current practice amongst Dutch gynaecologists and midwives. Midwifery.

[46] Hauck Y.L., Kelly G., Dragovic M., Butt J., Whittaker P., Badcock J.C. (2015). Australian midwives knowledge, attitude and perceived learning needs around perinatal mental health. Midwifery.

[47] Jones C.J., Creedy D.K., Gamble J.A. (2011). Australian Midwives’ Knowledge of Antenatal and Postpartum Depression: A National Survey. J. Midwifery Womens Health.

[48] Islam M.d.J., Broidy L., Baird K., Mazerolle P. (2017). Intimate partner violence around the time of pregnancy and postpartum depression: The experience of women of Bangladesh. PLoS ONE.

[49] Shakespeare J., Youd E., Knight M., MU Other Psychiatric and Homicide Chapter Writing Group (2018). Message for the care of women from vulnerable groups. Saving Lives, Improving Mothers’ Care-Lessons Learned to Inform Maternity Care from the UK and Ireland Confidential Enquiries into Maternal Deaths and Morbidity 2014–16.

[50] Ayre K., Gordon H.G., Dutta R., Hodsoll J., Howard L.M. (2019). The Prevalence and Correlates of Self-Harm in the Perinatal Period. J. Clin. Psychiatry..

[51] Cairns A., Kenyon S., Patel R., Bunch K., Knight M. (2022). on behalf of the MU mental health chapter writing group. Improving mental health care and care for women with multiple adversity. Saving Lives, Improving Mothers’ Care Core Report—Lessons learned to inform maternity care from the UK and Ireland Confidential Enquiries into Maternal Deaths and Morbidity 2018–2020.

[52] Darwin Z., Domoney J., Iles J., Bristow F., McLeish J., Sethna V. (2021). Involving and Supporting Partners and Other Family Members in Specialist Perinatal Mental Health Services: Good Practice Guide.

[53] Ramluggun P., Kamara A., Anjoyeb M. (2020). Postnatal depression in fathers: A quiet struggle?. Br. J. Ment. Health Nurs..

[54] Das R., Hodkinson P. (2019). Tapestries of Intimacy: Networked Intimacies and New Fathers’ Emotional Self-Disclosure of Mental Health Struggles. Soc. Media Soc..

[55] Anding J., Röhrle B., Grieshop M., Schücking B., Christiansen H. (2015). Early Detection of Postpartum Depressive Symptoms in Mothers and Fathers and Its Relation to Midwives’ Evaluation and Service Provision: A Community-Based Study. Front. Pediatr..

[56] Rominov H., Giallo R., Pilkington P.D., Whelan T.A. (2017). Midwives’ perceptions and experiences of engaging fathers in perinatal services. Women Birth.

[57] Mangla K., Hoffman M.C., Trumpff C., O’Grady S., Monk C. (2019). Maternal self-harm deaths: An unrecognized and preventable outcome. Am. J. Obstet. Gynecol..

[58] Hope H., Pierce M., Osam C.S., Morgan C., John A., Abel K.M. (2022). Self-harm risk in pregnancy: Recurrent-event survival analysis using UK primary care data. Br. J. Psychiatry.

[59] Williamson A.E., McQueenie R., Ellis D.A., McConnachie A., Wilson P. (2021). ‘Missingness’ in health care: Associations between hospital utilization and missed appointments in general practice. A retrospective cohort study. PLoS ONE.

[60] McQueenie R., Ellis D.A., McConnachie A., Wilson P., Williamson A.E. (2019). Morbidity, mortality and missed appointments in healthcare: A national retrospective data linkage study. BMC Med..

[61] Higgins A., Carroll M., Downes C., Monahan M., Gill A., Madden D. (2017). Perinatal Mental Health: An Exploration of Practices, Policies, Processes and Education Needs of Midwives and Nurses Within Maternity and Primary Care Services in Ireland.

[62] Fletcher A., Murphy M., Leahy-Warren P. (2021). Midwives’ experiences of caring for women’s emotional and mental well-being during pregnancy. J. Clin. Nurs..

[63] Magdalena C.D., Tamara W.K. (2020). Antenatal and postnatal depression—Are Polish midwives really ready for them?. Midwifery.

[64] McGookin A., Furber C., Smith D.M. (2017). Student midwives’ awareness, knowledge, and experiences of antenatal anxiety within clinical practice. J. Reprod. Infant. Psychol..

[65] Lau R., McCauley K., Barnfield J., Moss C., Cross W. (2015). Attitudes of midwives and maternal child health nurses towards suicide: A cross-sectional study. Int. J. Ment. Health Nurs..

